# Evaluation of the Effectiveness of Xanthine Oxidoreductase Inhibitors on Haemodialysis Patients using a Marginal Structural Model

**DOI:** 10.1038/s41598-017-13970-4

**Published:** 2017-10-25

**Authors:** Takeo Ishii, Masataka Taguri, Kouichi Tamura, Kunio Oyama

**Affiliations:** 1 Department of Internal Medicine, Yokohama City, Yokohama-Daiichi Hospital, Yokohama, 220-0011 Japan; 2 Department of Medical Science and Cardiorenal Medicine, Yokohama City University Graduate School of Medicine, Yokohama, 236-0004 Japan; 3 Department of Biostatistics, Yokohama City University Graduate School of Medicine, Yokohama, 236-0004 Japan

## Abstract

A lower serum uric acid (UA) level has been associated with a higher mortality rate in haemodialysis patients. We investigated the long-term confounding factors of UA and mortality, and fitted a marginal structural model (MSM) based on the causal effect of xanthine oxidoreductase inhibitors (XORi). In total, 2429 patients on regular dialysis from April 2013 to March 2016 were included, and divided into quintiles by serum UA with Kaplan Meier (KM) curves and log rank analysis. Baseline characteristics were evaluated for relationships with all-cause mortality and cardiovascular disease (CVD) using the Cox hazard model. The MSM was used to control for time-dependent confounders of the XORi treatment effect. KM curves indicated that patients in the highest UA quintile had better outcomes than those in the lowest UA quintile. UA was not correlated with all-cause mortality or CVD events in the Cox model; however, the hazard ratio (HR) for mortality was 0.96 for the baseline administration of XORi. The MSM analysis for the effect of XORi treatment on all-cause mortality revealed a HR of 0.24 (95% confidence interval: 0.15-0.38) in all cohorts. These results suggest that XORi improved all-cause mortality in end-stage renal disease, irrespective of the serum UA level.

## Introduction

In the general population, an elevated serum uric acid (UA) level or hyperuricaemia reportedly aggravates cardiovascular disease (CVD) and chronic kidney disease (CKD) progression. In nondialysis CKD patients, higher serum UA levels accelerate the deterioration of kidney function^1–4^. In addition, the results of several randomized trials conducted to examine the effect of the UA lowering agent allopurinol in CKD and/or hypertensive patients have been reported^5–7^. In these randomized clinical trials, the group allocated to allopurinol showed a slower progression of CKD^5–7^. In addition, further long-term follow-up after completion of these randomized trials showed that the treatment with allopurinol significantly slowed the progression of kidney disease and reduced cardiovascular risk^8^.

Several recent studies have reported that a lower serum UA in haemodialysis CKD patients was associated with unfavourable outcomes^9–11^ than in nondialysis CKD patients. The reason for these paradoxical findings, i.e. that a lower serum UA predicted unfavourable outcomes and a higher UA may even be favourable in haemodialysis patients, is unclear. In haemodialysis patients, although a relationship with malnutrition has been suggested^12^, the exact reason remains undetermined. While lower serum UA levels have been linked to unfavourable outcomes in haemodialysis patients, UA lowering agents continue to be administered frequently without solid supporting evidence. Investigations are needed to explore the relationships between UA and outcomes, and the effect of UA lowering treatments on mortality in haemodialysis patients.

An important clue to the elucidation of the mechanism underlying the UA lowering therapy-related beneficial effects may be its inhibitory effect on xanthine oxidoreductase (XOR). XOR is synthesized as a dehydrogenase (XDH) and is readily converted to its oxidase form xanthine oxidase (XO) by either proteolysis or modification of its cysteine residues^13^. Under ischaemic or inflammatory conditions, XDH is converted to XO, which then catalyses the oxidation of hypoxanthine to xanthine and then to UA, with consequent production of the superoxide anion (O_2_−) and hydrogen peroxide (H_2_O_2_)^13^. The newly developed drugs febuxostat and topiroxostat in Japan have been used in patients with CKD and haemodialysis patients with hyperuricaemia. Hosoya *et al*.^14^ reported the renal protective effect of topiroxostat with decreases in urine albumin excretion in patients with CKD, possibly due to the activity of XOR inhibitors (XORi) rather than the UA lowering effect.

The present study investigated the association of baseline UA, controlling for the confounding factors mortality and CVD events in patients treated in our haemodialysis centres over a period of three years. The treatment effects of allopurinol and febuxostat on mortality and CVD events were estimated using MSM analysis^15,16^.

## Results

**Figure 1:**
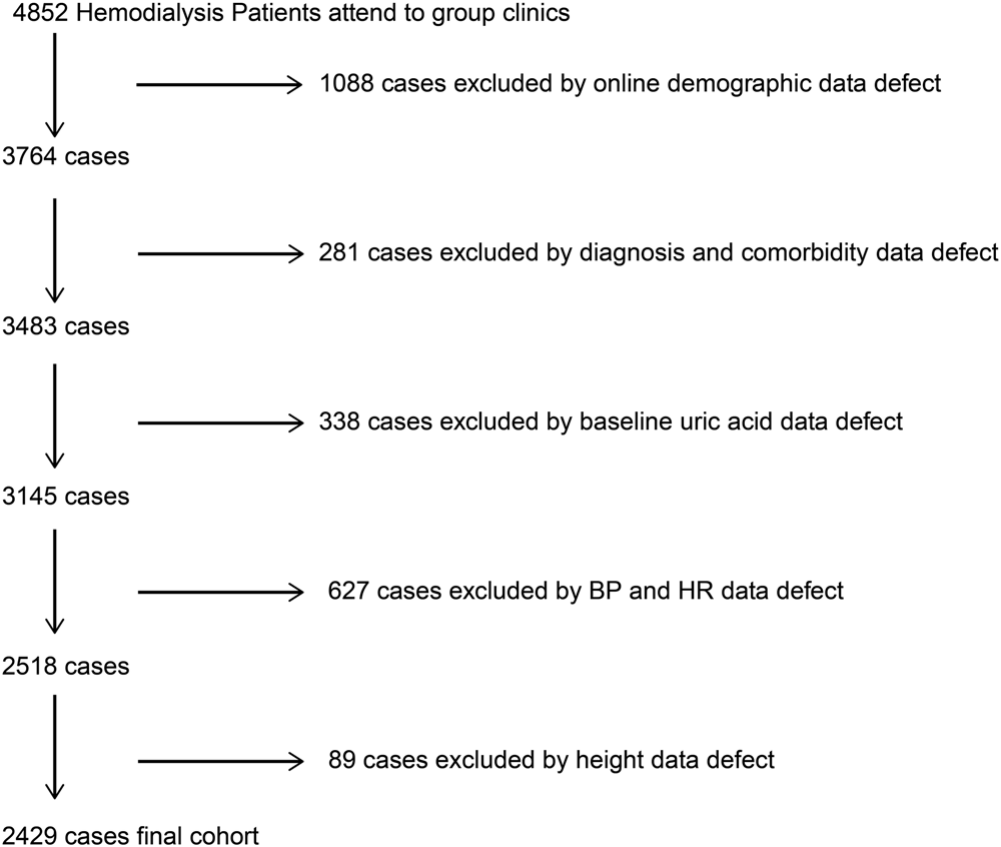
Study population selection from the Step II data system. We identified maintenance haemodialysis patients who attended our group’s clinics from April 2013 to March 2016; 2429 patients were finally included in the study cohort.

A total of 2429 haemodialysis patients were included in the study cohort (Fig. 1). Patients were divided into quintiles according to their baseline serum UA. The average serum UA level was 5.81 +/− 0.67 mg/dL in quintile 1, 6.61 mg/dL in quintile 2, 7.29 mg/dL in quintile 3, 7.84 mg/dL in quintile 4 and 9.29 mg/dL in quintile 5. XORi was administered in 470 (19.3%) cases at baseline, including 31.6% of patients in quintile 1, 22.8% in quintile 2, 15.7% in quintile 3, 13.3% in quintile 4, and 12.8% in quintile 5 (Table 1). Other laboratory findings and comorbidities are shown in Table 1. Of the original 2429 patients, 241 dropped out during the 3-year study period, while another 456 patients died. The total number of CVD events was 1278; heart failure occurred in 545, ischaemic heart disease in 375, atrial fibrillation and valvular disease in 150, new-onset hypertensive disease in 97, and other events were recorded in one patient. Some patients had multiple CVD events over the 3-year period.

**Table 1:** Baseline Characteristics.

Quintile	1			2			3			4			5			Total			ANOVA
n	493			482			535			435			484			2429			
Age (y)	70.0	±	11.7	68.7	±	12.1	68.2	±	12.0	66.4	±	12.4	63.2	±	12.7	67.3	±	12.4	P < 0.001
BMI (kg/m^2^)	21.4	±	3.5	21.6	±	3.4	21.4	±	3.8	22.1	±	3.9	22.6	±	4	21.8	±	3.8	p < 0.001
Sex, male n (%)	333		67.5	309		64.1	374		69.9	282		64.8	315		65.1	1613		66.4	p = 0.264
DM yes n (%)	238		48.3	224		46.5	223		41.7	161		37.0	142		29.3	988		40.7	p < 0.001
XORi, yes n (%)	156		31.6	110		22.8	84		15.7	58		13.3	62		12.8	470		19.3	p < 0.001
HD vintage (m)	74.3	±	99.7	75.1	±	79.4	71.1	±	73.7	74.7	±	76.9	78.4	±	88.8	74.6	±	84.2	NS
Hb (g/dL)	10.7	±	1.0	10.7	±	1.0	10.9	±	1.0	10.8	±	1.0	10.9	±	1.1	10.8	±	1.0	p < 0.001
Fe (µg/d)	68.3	±	23.1	70.2	±	21.9	71.2	±	22.4	71.3	±	21.8	74.1	±	27.7	71.0	±	23.5	p = 0.004
Ferritin (ng/mL)	92.2	±	92.7	98.0	±	96.1	101.9	±	107.7	104.3	±	122.6	88.4	±	142.5	96.9	±	113.5	p = 0.167
BUN (mg/dL)	56.6	±	13.8	60.1	±	13.7	62.7	±	13.8	66.6	±	14.7	71.6	±	14.3	63.4	±	15.0	p < 0.001
UA (mg/dL)	5.8	±	0.7	6.6	±	0.2	7.3	±	0.6	7.8	±	0.7	9.3	±	1.0	7.3	±	1.3	p < 0.001
Cr (g/dL)	9.0	±	2.6	9.6	±	2.6	9.9	±	2.6	10.2	±	2.8	11.0	±	2.8	9.9	±	2.8	p < 0.001
Na (mEq/L)	137.6	±	2.2	138.0	±	2.1	137.8	±	2.1	138.2	±	1.9	138.1	±	2.0	137.9	±	2.1	p = 0.001
Pi (mg/dL)	5.0	±	1.1	5.1	±	1.1	5.2	±	1.1	5.4	±	1.2	5.7	±	1.3	5.3	±	1.2	p < 0.001
Intact PTH (pg/mL)	178.5	±	118.8	187.2	±	151.4	206.4	±	161.0	203.6	±	178.6	216.6	±	172.2	198.5	±	157.8	p = 0.001
Alb (g/mL)	3.5	±	0.4	3.6	±	0.4	3.6	±	0.4	3.6	±	0.4	3.7	±	0.4	3.6	±	0.4	p < 0.001
B2MG (mg/dL)	27.0	±	6.5	27.6	±	6.5	27.6	±	6.5	28.1	±	7.2	28.3	±	6.9	27.7	±	6.7	p < 0.05
TC (mg/dL)	149.7	±	32.4	153.1	±	32.6	154.1	±	31.3	155.1	±	32.9	160.9	±	34.5	154.5	±	32.9	p < 0.001
LDL (mg/dL)	83.0	±	23.1	86.0	±	24.3	85.7	±	23.1	86.9	±	22.6	90.4	±	25.9	86.4	±	23.9	p < 0.001
HDL (mg/dL)	73.8	±	12.9	73.8	±	11.6	74.0	±	12.7	74.7	±	12.7	75.8	±	12.3	74.4	±	12.5	p = 0.067
CGR (%)	84.3	±	25.6	90.4	±	26.2	92.4	±	25.9	93.2	±	27.8	97.3	±	26.8	91.5	±	26.8	p < 0.001
Delta BW (kg/HD)	2.2	±	1.1	2.2	±	1.0	2.4	±	1.1	2.4	±	1.1	2.6	±	1.2	2.4	±	1.1	p < 0.001
SBP (mmHg)	156.7	±	25.8	157.1	±	25.5	156.3	±	23.3	157.2	±	25.1	153.2	±	24.9	156.1	±	24.9	p = 0.085
nPCR (g/kg/day)	0.80	±	0.16	0.84	±	0.15	0.87	±	0.16	0.90	±	0.17	0.95	±	0.18	0.87	±	0.17	p < 0.001
KT/V	1.4	±	0.3	1.4	±	0.3	1.4	±	0.3	1.4	±	0.3	1.4	±	0.3	1.4	±	0.3	p = 0.29
Comorbidity n (%)			(%)			(%)			(%)			(%)			(%)	Total		(%)	Pearson Chi Square
Infection and parasite	272		21.0	273		21.1	276		21.3	232		17.9	241		18.6	1,294		100	0.209
Neoplasms	25		19.4	29		22.5	32		24.8	23		17.8	20		15.5	129		100	0.672
Endocrine metabolic disorder	423		85.8	419		86.9	462		86.4	385		885.0	421		80.0	2110		86.9	0.801
Mental disorder	92		18.7	91		18.9	99		18.5	85		19.5	75		15.5	442		18.2	0.532
Nervous system disorder	255		18.8	292		21.6	301		22.2	248		18.3	258		19.1	1,354		100	0.801
Eye/ear disorder	151		19.0	152		19.1	190		23.9	152		19.1	150		18.9	795		100	0.532
CVD complications (baseline)	391		79.3	396		82.2	430		80.4	351		80.7	389		80.4	1957		80.6	0.561
Heart Failure	78		15.8	88		18.3	86		16.1	69		15.9	82		16.9	403		16.6	0.828
Hypertensive disease	359		72.8	376		78.0	406		75.9	329		75.6	365		75.4	1835		75.5	0.462
Ischaemic Heart Disease	176		35.7	162		33.6	183		34.2	128		29.4	146		30.2	795		32.7	0.179
Pulmonary Heart Diseases	0		0.2	2		0.4	0		0.0	2		0.5	0		0.0	5		0.2	0.343
Af and Valvular Diseases	37		7.5	44		9.1	49		9.2	36		8.3	46		9.5	212		8.7	0.804
Cerebrovascular Diseases	66		13.4	66		13.7	72		13.5	55		12.6	56		11.6	315		13.0	0.862
Diseases of the Arteries	132		26.8	112		23.2	109		20.4	98		22.5	84		17.4	535		22.0	0.007
Diseases of the Veins	27		5.5	35		7.3	32		6.0	27		6.2	25		5.2	146		6.0	0.694
Hypotension and Others	31		6.3	29		6.0	25		4.7	22		5.1	21		4.3	128		5.3	0.588
Respiratory system	393		19.7	400		20.1	434		21.8	364		18.3	401		20.1	1,992		100	0.307
Digestive system	413		20.0	411		19.9	449		21.8	377		18.3	413		20.0	2,063		100	0.841
Skin disorder	374		20.1	372		20.0	416		22.4	336		18.1	363		19.5	1,861		100	0.492
Musculoskeletal	372		20.2	367		20.0	409		22.3	323		17.6	367		20.0	1,838		100	0.724
PCKD and congenital disease	65		20.6	66		20.9	70		22.2	52		16.5	63		19.9	316		100	0.839
Injury	189		20.3	200		21.5	215		23.1	160		17.2	168		18.0	932		100	0.948

The average haemodialysis duration was 74.6 months; average BMI was 21.8 (kg/m^2). Diabetes was present in 40.7%. Patients were divided into quintiles by serum uric acid. The average serum UA was 5.81 +/− 0.67 mg/dL in quintile 1, 6.61 mg/dL in quintile 2, 7.29 mg/dL in quintile 3, 7.84 mg/dL in quintile 4 and 9.29 mg/dL in quintile 5. A XOR inhibitor was administered in 470 cases at baseline (19.3%). In quintile 1 31.6%, quintile 2 22.8%, quintile 3 15.7%, quintile 4 13.3%, quintile 5 12.8%, respectively, of the baseline administration rate for XORi. Abbreviations: Alb, albumin; B2MG, beta-2 microglobulin; BMI, body mass index; BUN, blood urea nitrogen; BW, body weight; CGR, creatinine generation rate; Cr, creatinine; DM, diabetes mellitus; Fe, iron; Hb, haemoglobin; KT/V, urea adequacy measure scales dialysis dose; LDL, low-density lipoprotein; Na, sodium; nPCR, normalised protein catabolic rate; Pi, serum phosphate; PTH, parathyroid hormone; SBP, systolic blood pressure; UA, uric acid; XOR, xanthine oxidoreductase; XORi, xanthine oxidoreductase inhibitors; and PCKD, polycystic kidney disease.

### Kaplan Meier Curves and Log Rank Analysis

**Figure 2:**
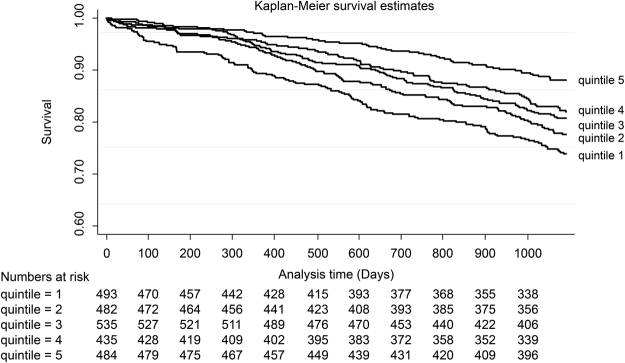
Kaplan Meier survival curves of all cohort patients. Kaplan Meier survival curves indicated that quintile 5 had a significantly more favourable outcome than quintile 1. Log rank analysis was performed between the quintiles. Quintile 5 had a significantly higher survival time than all other quintiles.

Kaplan Meier survival curves indicated that the patients in quintile 5 had a significantly more favourable outcome than those in quintile 1. Over the 3 years, 456 (18.8%) patients died in all quintiles. In quintile 1, 123/493 (24.9%) died, while 55/484 (11.4%) died in quintile 5 (Fig. 2). Log rank analysis for CVD events was not significant.

### Cox Hazard Analysis

**Figure 3:**
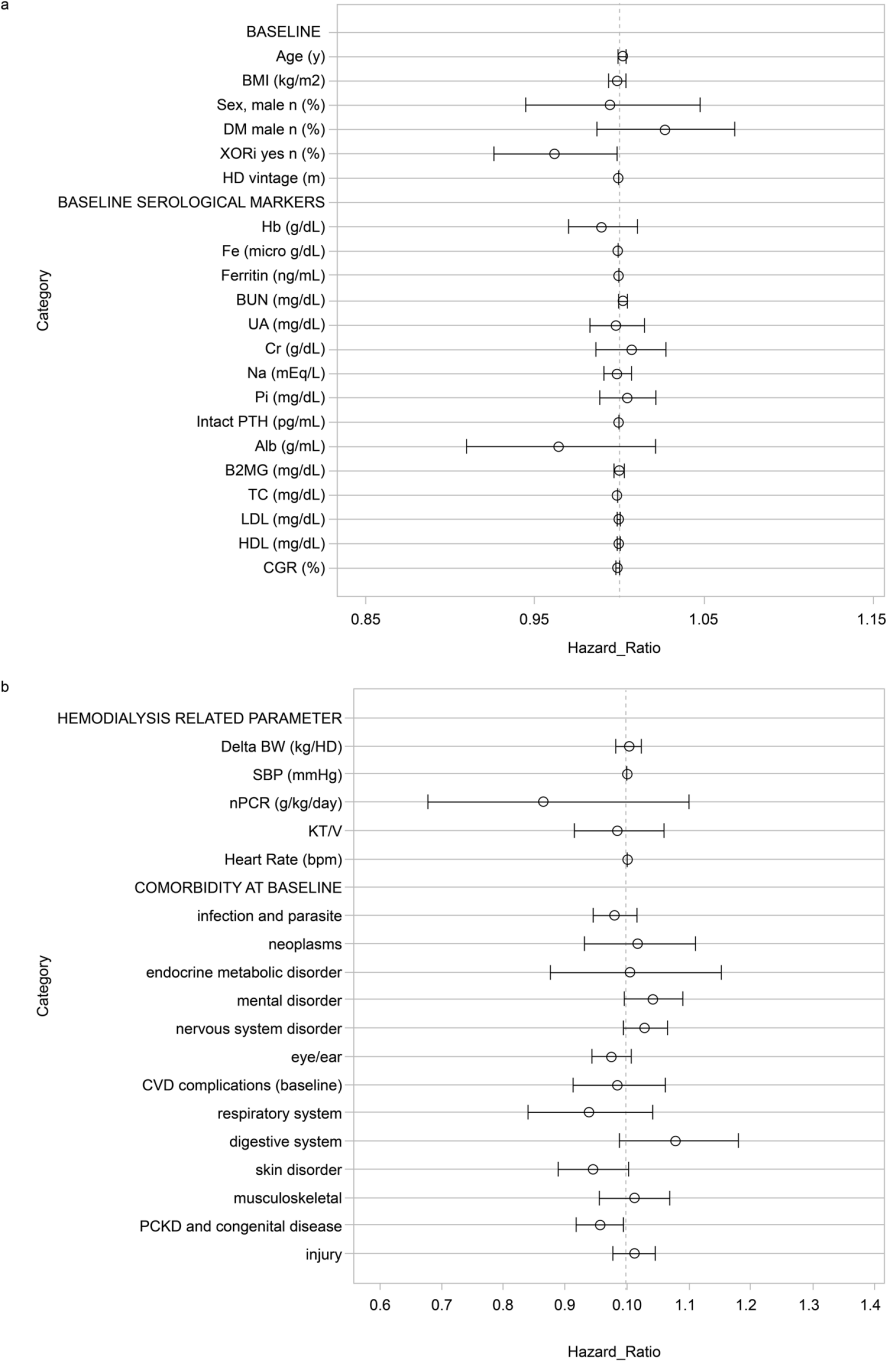
Cox Hazard analysis for all-cause mortality (multivariate a and b). (**a**) Estimated correlation between baseline characteristics and mortality (demographic data to serological markers). (**b**) Estimated correlation between baseline comorbidities and all-cause mortality. The HR of baseline XORi administration for all-cause mortality was 0.962 (95% confidence interval [CI] 0.926–0.999) and the baseline PCKD and congenital disease 0.956 (95% CI 0.918–0.996).

We performed Cox hazard analysis to estimate the correlation between the baseline characteristics, mortality and CVD events. The baseline UA level was not significantly correlated with all-cause mortality or CVD events (see Table 2, and Fig. 3a,b). The administration of XORi was associated with an HR of 0.96 (95% confidence interval [CI] 0.926–0.999) (Table 2) for all-cause mortality, but it was not associated with CVD events. The HR of the baseline PCKD and congenital disease for all-cause mortality was 0.96 (95% CI 0.918–0.996). CVD events were not correlated with the baseline administration of XORi; however, they were correlated with baseline diabetes (HR1.072 [95% CI 1.023–1.124]), musculoskeletal complications (HR 1.06 [95% CI 1.005–1.119]), and injury (HR 1.05 [95% CI 1.054–1.107]) (Supplementary Table S1).

**Table 2:** Cox Hazard Analysis for All-Cause Mortality.

Parameter	Hazard Ratio	SE	p	95% CI		*p < 0.05
				Lower Limit	Upper Limit	
**Baseline**						
Age (y)	1.00	0.0011	0.06	1.00	1.00	
BMI (kg/m^2^)	1.00	0.0026	0.62	0.99	1.00	
Sex, male n (%)	1.00	0.026	0.84	0.95	1.05	
DM yes n (%)	1.03	0.020	0.19	0.99	1.07	
XORi, yes n (%)	0.96	0.019	0.05	0.93	1.00	*
HD vintage (m)	1.00	0.00011	0.51	1.00	1.00	
**Baseline Serological Parameters**						
Hb (g/dL)	0.99	0.010	0.34	0.97	1.01	
Fe (µg/dL)	1.00	0.00039	0.09	1.00	1.00	
Ferritin (ng/mL)	1.00	0.000086	0.15	1.00	1.00	
BUN (mg/dL)	1.00	0.0013	0.11	1.00	1.01	
UA (mg/dL)	1.00	0.0082	0.85	0.98	1.02	
Cr (g/dL)	1.01	0.011	0.53	0.99	1.03	
Na (mEq/L)	1.00	0.0041	0.73	0.99	1.01	
Pi (mg/dL)	1.01	0.0082	0.51	0.99	1.02	
Intact PTH (pg/mL)	1.00	0.000039	0.07	1.00	1.00	
Alb (g/mL)	0.96	0.030	0.22	0.91	1.02	
B2MG (mg/dL)	1.00	0.0016	0.93	1.00	1.00	
TC (mg/dL)	1.00	0.00037	0.11	1.00	1.00	
LDL (mg/dL)	1.00	0.00045	0.96	1.00	1.00	
HDL (mg/dL)	1.00	0.00054	0.97	1.00	1.00	
CGR (%)	1.00	0.00094	0.51	1.00	1.00	
**Hemodialysis Related Parameters**						
Delta BW (kg/HD)	1.00	0.011	0.86	0.98	1.02	
SBP (mmHg)	1.00	0.00035	0.98	1.00	1.00	
nPCR (g/kg/day)	0.86	0.12	0.23	0.68	1.10	
KT/V	0.99	0.038	0.68	0.92	1.06	
Heart Rate (bpm)	1.00	0.00072	0.97	1.00	1.00	
**Comorbidity at Baseline**						
Infection and parasite	0.98	0.018	0.27	0.95	1.02	
Neoplasms	1.02	0.045	0.71	0.93	1.11	
Endocrine metabolic disorder	1.01	0.070	0.94	0.88	1.15	
Mental disorder	1.04	0.023	0.08	1.00	1.09	
Nervous system disorder	1.03	0.018	0.11	0.99	1.07	
Eye/ear	0.98	0.017	0.14	0.94	1.01	
CVD complications (baseline)	0.98	0.039	0.68	0.91	1.06	
Respiratory system	0.94	0.055	0.23	0.84	1.04	
Digestive system	1.08	0.045	0.09	0.99	1.18	
Skin disorder	0.95	0.031	0.06	0.89	1.00	
Musculoskeletal	1.01	0.028	0.68	0.96	1.07	
PCKD and congenital disease	0.96	0.021	0.03	0.92	1.00	*
Injury	1.01	0.018	0.52	0.98	1.05	

Baseline XORi administration had a preventive effect on all-cause mortality (HR 0.962). CVD complications (baseline) include: heart failure, hypertensive disease, ischaemic heart disease, pulmonary heart diseases, af and valvular disease, cerebrovascular diseases, diseases of the arteries, diseases of the veins, hypotension and others. *Estimated correlation between baseline characteristics and mortality and CVD events (Supplementary Table S2). XORi treatments at baseline were associated with mortality. Abbreviations: Alb, albumin; B2MG, beta-2 microglobulin; BUN, blood urea nitrogen; BMI, body mass index; BW, body weight; Cr, creatinine; DM, diabetes mellitus; Fe, iron; Hb, haemoglobin; HD, haemodialysis; hdl, high-density lipoprotein; hr, heart rate; KT/V, urea adequacy measure scales dialysis dose; LDL, low-density lipoprotein; nPCR, normalised protein catabolic rate; Pi, phosphate; PTH, parathyroid hormone; SBP, systolic blood pressure; tc, total cholesterol; UA, uric acid; XORi, xanthine oxidoreductase inhibitors; af, atrial fibrillation; and PCKD, polycystic kidney disease.

### Marginal Structural Model Analysis

**Figure 4:**
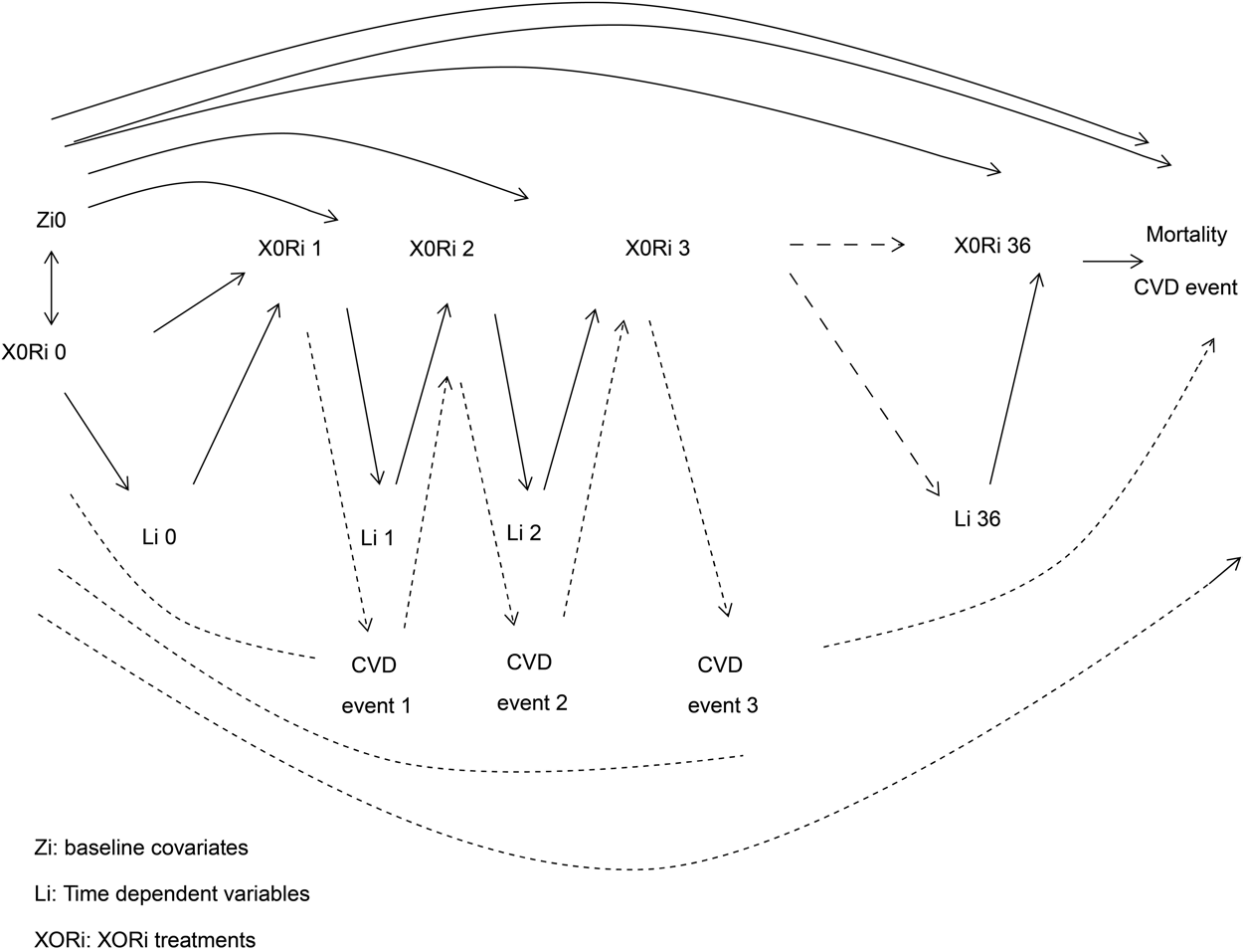
Marginal structural model (MSM) causal diagram for all-cause mortality and cardiovascular disease events. Xanthine oxidoreductase inhibitors (XORi) at baseline were included in the time non-dependent covariates and influenced the study period, but the presence or absence of XORi treatment was included in the MSM analysis as a time dependent variable. Previous treatments were used for IPTW analysis.

We utilised a marginal structural model (MSM) for this longitudinal repeated measure analysis to estimate the treatment effect of XORi in haemodialysis patients according to the method of Robins and Hernan^15,16^ (Fig. 4). For analysis of the treatment propensity score, the factors strongly influencing treatment choice as determined by logistic analysis are shown in Supplementary Table S2. Treatment selection was influenced by age, BMI, endocrine metabolic disorders, mental disorders, nervous system disorders, eye/ear complications, diseases of the veins, hypotension, respiratory system and digestive system complications, musculoskeletal and injury at baseline complications, previous XORi, previous UA level, previous albumin, previous normalized protein catabolic rate (nPCR), previous creatinine generation rate (CGR), previous ferritin level, previous serum sodium level and previous dialysis adequacy (KT/V). After establishing the 1/denominator (1/ps_pred_n_, 1/(1-ps_pred_n_) and numerator (ps_pred_n_, 1-ps_pred_n_) (Supplementary Figure S1, Supplementary Table S3), the calculated stabilized weight for treatment was defined as follows. The mean stabilized weight was 1.11, the standard deviation was 0.58 (minimum 0.0089, maximum 21.95), the 95% point was 1.94, and the 99% point was 3.62 (Supplementary Table S3).

**Figure 5:**
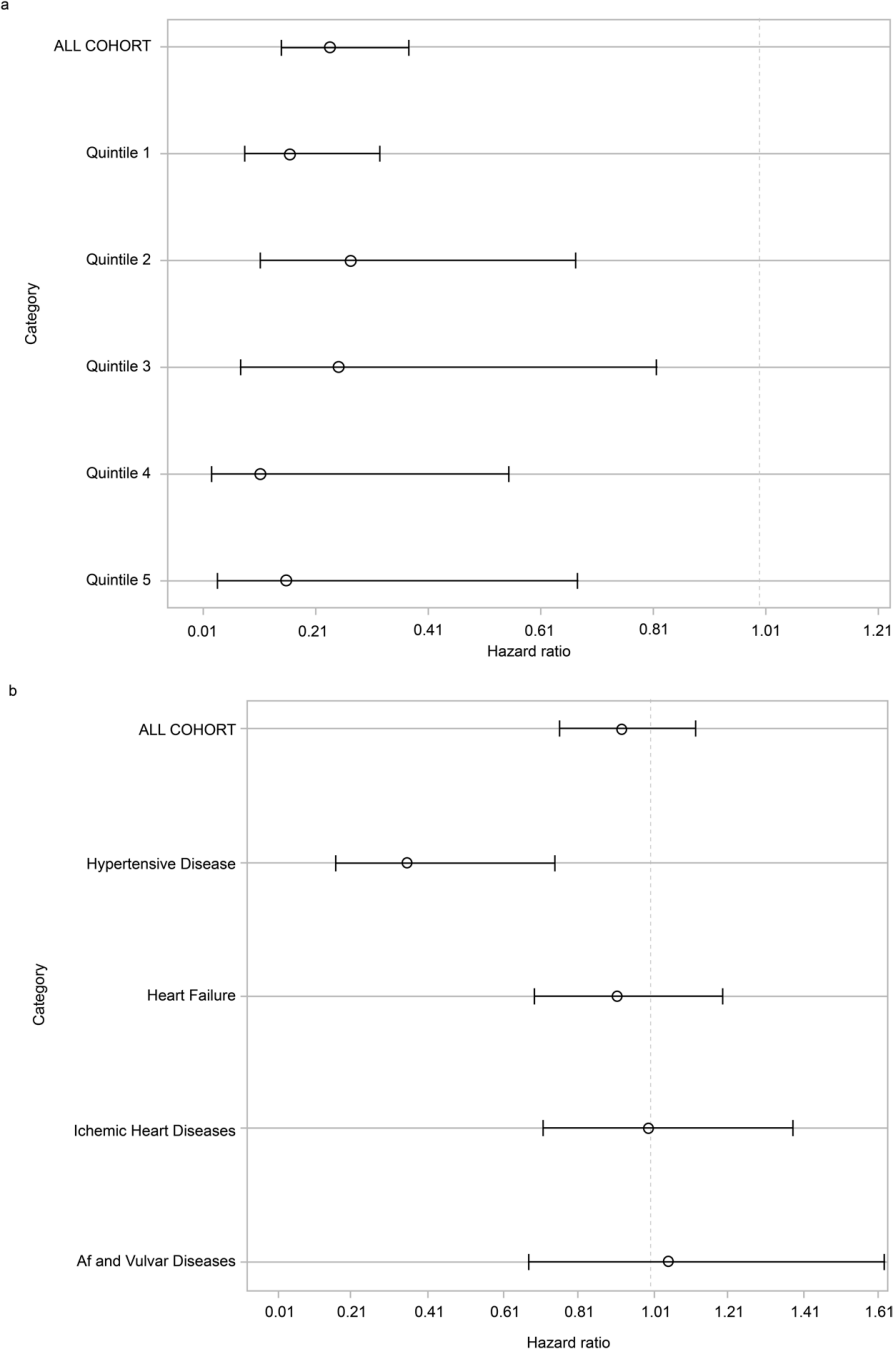
Marginal Structural Model (MSM) Analysis of the Treatment Effect. (**a**) MSM for all-cause mortality. Treatment effect of Xanthine oxidoreductase inhibitors (XORi) estimated by the MSM for all-cause mortality was estimated to have an HR of 0.24 (95% confidence interval [CI] 0.15–0.38) in all cohorts. The quintile-1 HR was 0.17, quintile-2 HR 0.27, quintile-3 HR 0.25, quintile-4 HR 0.11 and quintile-5 HR 0.16. (**b**) MSM for CVD events. The treatment effect of XORi on CVD events was associated with a HR of 0.92 (95% CI 0.76–1.12) in all quintiles. The treatment effect for hypertensive disease included in the CVD events indicated a significantly preventive effect with a HR of 0.35 (95% CI 0.16–0.75). The HR was 0.91, 0.997, 1.05 and 1.05 in heart failure, ischaemic heart disease, atrial fibrillation (af) and valvular disease, respectively. CVD events other than hypertensive disease, heart failure, ischaemic heart disease, af and vulvar disease, could not be calculated in MSM analysis due to the small number of outcomes.

The treatment effect of the XOR inhibitors was estimated by MSM utilizing stabilized weight for all-cause mortality was associated with an HR of 0.24 (95% CI 0.15–0.38) in all of the quintiles (Fig. 5a). The quintile-1 HR was 0.17 (95% CI 0.085–0.32), quintile-2 HR 0.27 (95% CI 0.11–0.67), quintile-3 HR 0.25 (95% CI 0.077–0.82), quintile-4 HR 0.11 (95% CI 0.023–0.56) and quintile-5 HR 0.16 (95% CI 0.037–0.67). The treatment effect of XORi on CVD events was associated with an HR of 0.92 (95% CI 0.76–1.12) in all of the quintiles (Fig. 5b). Nevertheless, the treatment effect of XORi for hypertensive disease included in the CVD events indicated a significantly preventive effect, with an HR of 0.35 (95% CI 0.16–0.75) (Fig. 5b).

Sensitivity analysis was performed via MSM, since a small number of patients with unusual prescription patterns had a large effect on the analysis after multiplying by 36 visits. The stabilized weight was truncated after the 95^th^ percentile (0.69–1.94) or 99^th^ percentile (0.26–3.62) (Supplementary Table S3). After truncation at each level, the weights were reset to each point level (e.g., weights <0.26 were reset to 0.26; otherwise, weights >3.62 were reset to 3.62)^15–17^. The 99^th^ percentile truncated MSM analysis of HR for all-cause mortality was found to be 0.24 (95% CI 0.152–0.370). The 95^th^ percentile truncated MSM analysis of HR for all-cause mortality was found to be 0.24 (95% CI 0.161–0.368). The HR for CVD events with 95^th^ percentile weight analysis was 0.91 (95% CI 0.770–1.065). The HR for new onset hypertensive diseases with 95^th^ percentile weight analysis was 0.45 (95% CI 0.215–0.939), with a 99^th^ percentile of 0.40 (95% CI 0.190–0.843).

## Discussion

We used observational MSM to estimate HR of 0.24 for all-cause mortality in patients receiving XORi in this 3-year follow up study. Chen *et al*.^18^ reported that patients with gout treated with UA-lowering agents for over 2 years had a lower risk of all-cause mortality (HR 0.39 for allopurinol, 0.24 for benzbromarone) in a matched cohort in Taiwan. The results of an analysis conducted in Taiwan were similar to the results obtained in our study, in which MSM was applied. MSM was used to analyse repeatedly measured data; each visit and each patient was adjusted by using inverse probability of treatment weighted (IPTW) estimates with 2429 cases and 36 visits.

Kaplan Meier curves and log rank analysis showed that the lower quintile of UA exhibited a less favourable outcome than the higher quintile, but Cox hazard analysis showed that the UA level at baseline was not correlated with all-cause mortality or CVD events. Cox hazard analysis also indicated a preventive effect of baseline XORi administration on mortality (HR 0.962, 95% CI 0.926–0.999).

With regards to the other well-known confounders in haemodialysis, only XORi treatment at baseline was correlated with the outcomes. However, if other covariates such as certain well-known confounders (e.g., Alb, Hb, KT/V) were estimated as time dependent, the HR of these covariates would be different from the baseline Cox model. Thus, further investigation with a time dependent model is needed. The HR of 0.962 for XORi administration was estimated only at baseline and would likely change with different treatment patterns as a result of the cumulative treatment effects over the period of 3 years in this time-dependent repeated measured MSM analysis. A lower serum UA level in haemodialysis was correlated with unfavourable outcomes, but was not identified as the main cause of mortality in the Cox model, further suggesting that the therapeutic target might be the underlying high XO status caused by uraemia, hypertension, hypoxia or chronic inflammatory diseases. While Beberashviliet *et al*. reported an all-cause mortality rate of 43.5% in their dialysis patients^9^, our study showed a death rate of 16.5%. This difference in mortality may be related to the high-quality management of dialysis patients in Japan^19^. Beberashviliet *et al*. also reported an HR of 0.89 for all-cause mortality with serum uric acid levels in haemodialysis patients in a stepwise method using an adjusted Cox model^11^. However, our result using a fully adjusted Cox model did not show a significant correlation with the outcome.

Since UA is rapidly removed from the patients during each dialysis session, serum UA level itself should not be considered as an appropriate hyperuricaemia marker in haemodialysis CKD patient. Alternatively, from the results in the present study, it may be recommended that dialysis patients be treated with XORi to prevent tissue damage via inhibition of tissue XO activity irrespective of the serum UA level. Ives *et al*. reported that XO-derived reactive oxygen species (ROS), not UA, are the trigger for IL1β and also showed that XORi reduces ROS^20^. Macrophage secretion of IL1β is rapidly mediated by XOR activation, while XOR inhibition impairs IL1β secretion^20^. Yisireyili *et al*. reported that febuxostat decreased ROS production (H_2_O_2_, 8-OHdG and lipid peroxidation) and visceral adipose tissue inflammation in stress-induced hyperuricaemic mice having high levels of XOR^21^.

In contrast to the results obtained in a dialysis patient population, those obtained in a healthy normotensive population showed that higher UA levels were related to unfavourable outcomes in large-cohort observational studies^22,23^. However, considering the antioxidative effect of UA, a low UA level may even be unfavourable^24^. Since UA reportedly reduces oxidative stress and is suggested to prolong life span^24^, it appears that lowering the serum UA may paradoxically reduce the beneficial antioxidative effect of UA. Beberashvili *et al*. reported that a lower serum UA level was correlated with lower geriatric nutritional risk index scores^11^. We consider serum UA to be a superficial nutritional marker along with serum albumin and creatinine in haemodialysis CKD patients, and that the UA level should be relatively higher in haemodialysis patients. Higher UA levels were associated with better outcomes than lower levels in haemodialysis CKD patients^10,11^. However, achieving the therapeutic target of a lower UA would be due to the effects of the XORi rather than nutritional deficits. The optimal level of UA in haemodialysis is controversial. In the results of the present study, all UA quintiles showed a strong treatment effect (Fig. 5a) along with a protective effect for new-onset hypertensive diseases (Fig. 5b). We suggest that XORi administration in haemodialysis patients with higher UA levels along with those with lower UA levels improved outcomes.

XOR-derived free radicals may cause endothelial dysfunction and vasoconstriction by lowering nitric oxide availability and stimulating the upregulation of the renin-angiotensin system, which plays a pivotal role in inflammatory signalling^25^. Our MSM analysis indicated that XOR inhibition reduced the likelihood of the hypertensive diseases, suggesting that XOR inhibition acts directly on the vascular membrane. Through the activation of ROS, membrane damage, tissue hypoxia and endothelial dysfunction, hyperuricaemia is linked to or even provokes hypertension and coronary artery disease^26^. Our study did not find a reduction in new-onset ischaemic heart disease occurrence, but did find a preventive effect of XORi treatment against hypertensive diseases, which was be associated with stimulated NO production via XOR inhibitor-mediated suppression of oxidative stress^25^ (Fig. 5b).

Gondowin *et al*. reported that serum XOR activity was inversely related to Vitamin C and the oxidative enzyme xanthine oxidase, and serum XOR activity was increased in haemodialysis CKD patients compared with nondialysis CKD patients^27^. Nevertheless, the serum UA level was reportedly decreased in haemodialysis CKD patients compared with that in nondialysis CKD patients^27^. These findings suggest that the main determinant of oxidative stress production is xanthine oxidase activity, rather than the serum UA level, in both nondialysis and haemodialysis CKD patients. Mervaala *et al*. previously examined whether XOR produces the ROS, which are involved in the onset of angiotensin II (Ang II)-induced vascular dysfunction in double transgenic rats, and showed that Ang II–induced endothelial dysfunction was indeed associated with increased oxidative stress and vascular XO activity^28^. Laakso *et al*. reported that renal XOR activation occurred during the development of hypertension, with the ameliorating effects of a XORi elicited via its effect on NO synthesis in spontaneously hypertensive rats^29^.

These results from animal studies suggest that, together with progressive purine metabolism, the activation of tissue RAS and CKD progression may be a trigger that transforms XDH to XO in an oxidative-stress inducible form, and thus causes tissue damage. Since haemodialysis with residual renal function might be carried over to RAS activation, it is suggested that one cause of high XO is RAS activation; other causes include uraemia, hypoxia, and chronic inflammation. Additionally, XORi may have the ability to cut off the pathway from XO to tissue damage along with its effect on purine hypermetabolism.

It is expected that the administration of XORi in haemodialysis CKD patients, particularly those with high XO, would improve NO synthesis and exert ameliorating effects on the endothelial dysfunction evoked by oxidative stress and thus prevent tissue damage^30^. The present investigation of haemodialysis patients found that serum UA level was not an independent risk factor for all-cause mortality in the Cox model, but XORi was correlated with mortality, with a minor difference at baseline. It appears that the higher UA group among the haemodialysis CKD patients had a CVD event risk similar to that among patients with hyperuricaemia in the nondialysis population^18,31^. Nevertheless, the haemodialysis CKD patients with a wide range of serum UA levels were administered XORi in the present study, and the preventive effect on outcomes is likely to be due to the beneficial effect of XOR inhibition, even in the relatively lower UA group.

We presume that the high quintile group was exposed to a high oxidative stress environment along with an insufficient XORi dose in the higher UA group in this cohort (Table 1). As described in the study by Gondowin^27^, the serum XOR concentration in the haemodialysis CKD patients was higher than that in nondialysis CKD patients. We also presume that a higher administration rate of XORi in quintiles 4 and 5 (i.e., higher serum UA levels) would have a more favourable effect. In haemodialysis, a large amount of UA is removed, which is why attacks of gout are very rare. However, it is suggested that the underlying high XO status would be at the same level or higher as the hyperuricaemia in patients with CKD^23^. If UA were not removed during haemodialysis, the serum UA would be very high, with an accompanying attack of gout. A high XO should be presumed and XORi prescribed when high XO is suspected. Norman *et al*.^32^ suggested that a 600-mg/day dose of allopurinol should be considered for patients with stable angina; this may be a sufficiently high dose for patients on haemodialysis, because of the accumulation and urinary excretion limit of allopurinol.

In our cohort, patients in the higher quintiles seemed to have an insufficient dose of XORi; however, the effect of XOR inhibition appeared to be sufficient in all quintiles, even in the lower quintiles (Fig. 5a). Therefore, it is difficult to determine the difference in the efficacy of XOR inhibition between the quintiles in the present study. Further investigation is needed regarding treatment with newer agents such as topiroxostat. The recommendation from the present study is that a similarly high dose of XORi should be administered in the high and low UA level groups in cases of enhanced oxidative stress production due to malnutrition or chronic inflammatory status, as well as cases of severe lower leg arteriosclerosis or aspiration pneumonia. From the results presented here, it is recommended that XORi administration be initiated even when the serum UA concentration is approximately 6.0 mg/dL. In haemodialysis CKD patients, it has been recommended to lower the serum UA level to the same target level as nondialysis CKD patients to exert the anti-inflammatory effect mediated through its XOR inhibition. Concerning this important issue, the present study evaluated the effects of XOR inhibition, rather than the effect of UA lowering, on mortality due to CVD events.

There are several limitations of this study. First, we evaluated only the administration of XORi and not the dosage of each drug. However, the optimal dosage in haemodialysis patients has not been established. Therefore, we evaluated whether patients were prescribed XORi to evaluate the treatment effect in our statistical analysis. Second, we were not able to collect information about the cause of death after censoring. Information on cause of death in regularly visiting patients was immediately recorded in the system, and, after censoring we collected the outcome information as soon as possible. The cause of death was speculated to be the effect on CVD events and that XOR inhibition also influenced the rate of CV-related death. The third limitation was that combinations of drugs were not included in this analysis design; however, these drugs likely influenced serological markers, and, as in other published randomized trials, bias resulting from combinations of drugs could not be excluded. Fourth, we did not collect data on the incidence of XORi adverse events of XORi, but no serious adverse events were identified in the electronic records. Fifth, new-onset CVD events were derived from diagnoses in connection with claims data. However, the diagnosis was linked with a clinical diagnosis. Hypertensive diseases were defined as worsening of existing hypertension or new onset hypertension with the prescription of anti-hypertensive agents. Heart failure was diagnosed as NYHA II and greater according to the International Classification of Diseases, Tenth Edition (ICD-10), in this large sample. Sixth, there was a negative correlation between treatment and the serum UA level in the propensity score analysis for IPTW; however, this analysis was retrospective and the guideline for hyperuricaemia was not clearly defined. This is because recently reported evidence based on our Kaplan Meier curves showed that a higher UA level leads to more favourable outcomes than lower levels. We deduced that this reverse correlation of treatments and serum UA in IPTW creation was the result of adjustment for unusual XORi prescription patterns, such as the lower administration rate in the higher UA group. Table 1 shows a 12.8% rate of XORi prescription in quintile 5 at baseline and a 13.3% rate in the quintile 4 group, whereas rates of 31.6% and 22.8% are evident in quintile 1 and quintile 2, respectively. However, the negative correlation between XORi treatment and UA in the IPTW analysis is reflected in this prescription pattern. Further investigation is needed regarding this adjustment in future studies.

In conclusion, XOR inhibition was estimated in this repeated measured analysis to improve all-cause mortality and CVD events in end-stage renal disease patients on haemodialysis. The true therapeutic goal of treatment with XORi is suggested to be control of XOR activity rather than control of the serum UA level.

## Methods

### Study Population

The patient population consisted of 4852 haemodialysis patients treated in our group clinics. Cases were excluded based on insufficient demographic data (1088 cases), incomplete diagnosis and comorbidity data (281 cases), no baseline uric acid data (338 cases), incomplete data on BP and HR (627 cases) and lack of information on height (89 cases). Ultimately, this study included 2429 haemodialysis patients who were outpatients treated in 30 haemodialysis clinics in Yokohama Japan, between April 1, 2013 and March 31, 2016 (Fig. 1).

### Data Sources

The dialysis information system (STEP II®) includes information on the demographic data and outcomes, including information on the cause of death, and the STEPII^®^ system links to a laboratory data system and prescription data system. The start dates of the diagnosis and comorbidities were derived from the medical account processing system. The diagnoses and comorbidities were captured as ICD-10 codes along with the starting date.

### Outcomes

The primary outcome of this study was all-cause mortality. The secondary outcome was a CVD event (ICD-10 codes I00.0-I99.0) from the World Health Organization ICD-10. Hypertensive diseases were defined by worsening of hypertension, defined as a worsening of existing hypertension, or new onset of hypertension with the prescription of anti-hypertensive agents, including angiotensin-converting enzyme inhibitors, calcium channel blockers, Ang II receptor blockers, α-blockers, β-blockers and diuretics. Censored data were recorded when patients changed the clinic from one of our group clinics to another, underwent kidney transplantation, or were relocated from Yokohama. This study was conducted in accordance with the Declaration of Helsinki. The Review Board of Yokohama City University (A160324033) and Yokohama Daiichi Hospital approved this study. No consent was obtained as this study was retrospective in nature.

### XOR Inhibitor Exposure

The Guidelines of the Japanese Society of Hemodialysis, the Japanese Society of Gout and Nucleic Acid Metabolism, and the Japan Ministry of Health, Labour and Welfare recommend 50 mg/day allopurinol, 20 mg/day febuxostat, or 20 mg/day topiroxostat in patients with CKD on haemodialysis. We recorded whether patients used XORi and defined prescription treatment as binary (1 = true, 0 = false) for the statistical analysis. The prescription information was obtained from the STEP II® system. We prescribe once every two weeks for each patient and this information is recorded automatically into the STEP II® system along with the amount of each drug. We were able to define the prescription information at each visit. We converted these data into binary data.

### Statistical Analysis

#### Sample Size

Based on a previous report^18^ that showed that uric acid lowering treatment improved the mortality rate to 4.63% compared with 9.01% in untreated patients, the mortality rate ratio was 0.51 and the HR was 0.47 (0.29–0.79). Our null hypothesis was that there would be no difference in mortality in patients with and without XORi treatment. If the alpha error were 0.05 and the beta error 0.2, the study would require 913 cases in each group and 1826 cases in total. Our study satisfied the required number of cases with a cohort of 2429 cases.

We stratified all patients into quintile groups by the baseline serum UA level, and then compared differences in the baseline demographic data and serological markers using the chi-square test and ANOVA analysis. We described Kaplan Meier curves and used log rank analysis for UA quintiles at baseline. We fitted the Cox proportional hazard model for the mortality and CVD outcomes. Covariates were the demographic and comorbidity data along with the baseline serological markers. Along with numerous covariates for the Cox model, we fitted a robust estimate of the standard error in the SAS procedure^33^.

#### Marginal structural model

We created a marginal structural model (MSM) to estimate the treatment effect of XORi on mortality and CVD events, reasoning that prescribing decisions are influenced by serological parameters from the previous visit and past prescription patterns^15^. To adjust for the confounding effect of the prescribing of XORi, we calculated the inverse probability of treatment weighted (**IPTW**) estimates for XOR inhibition at each monthly visit, which in total was 36 times for each patient. Treatment was defined as binary, because allopurinol and febuxostat were both used in individual patients with hyperuricaemia. To the best of our knowledge, no study has compared data the treatment effect of these two drugs in haemodialysis patients.

In the MSM, we estimated the risk of death and CVD events between the treatment and non-treatment groups along with the confounders, and adjusted by cases and all visits. Weights were calculated by the method of Robins and Hernan *et al*.^15,16^. The dependent variable in the logistic regression was XORi use, while the covariates were the previous prescription binary data, time-independent demographic data, comorbidities at baseline and time-dependent serological markers that were the same as those used in the Cox hazard model for stabilized weight. Multivariate logistic regression was used to calculate treatment probabilities. Time-dependent covariates of past treatments and non-dependent covariates were used to calculate the treatment probabilities (propensity-score predicted: ps_pred) by logistic regression. The time-independent and time-dependent variables for logistic regression are shown in Supplementary Table S2.

The (propensity score for weight (IPTW,) IPTW (ps_weight) was created using the following formula: for patients treated with XORi, ps_weight = 1/ps_pred, otherwise 1/(1-ps_pred)^15,16^. The ps_weight was cumulatively multiplied by 36 visits, defined as 1/denominator. The numerator was created with the same covariate as the denominator, including previous XORi treatment, but without the time-dependent variables, and was created using the following formula: for patients treated with XORi, ps_weight = ps_pred, otherwise (1-ps_pred)^15,16^ (Supplementary Table S2). The numerator was cumulatively multiplied by 36 times, as was the denominator. The numerator and 1/denominator were multiplied to calculate the stabilized weight (numerator/denominator). The stabilized weight that was used to fit the outcome model of the MSM consisted of treatment at each visit and the outcome with stabilized weight^15,16^. In addition to all of the cohort MSM analyses, we performed MSM estimation for each quintile for mortality and CVD events. In the sub-analysis with the UA quintiles and elements of CVD events (such as hypertension, ischaemic heart disease, or heart failure), we adopted the polynomial month variable in the MSM analysis for the uric acid quintile. In the case of a small number of outcome events, we included the multiplied (month) and (month)^2^ and (month)^3^ variable, instead of the “month” variable, in the “MODEL” code in SAS^®^.

Missing data were replaced by the method of the last observation carried forward. The percent of missing data was within 8.0% in all observations, except for ferritin (9.5%), total cholesterol (8.2%) and systolic blood pressure before dialysis (8.3%). All statistical analyses were performed with SAS 9.3^®^ (SAS Corp.).

### Data availability

All data generated or analysed during this study are included in this published article (and its Supplementary Information files).

## Electronic supplementary material

Supplementary Information
