# Supplementary material for: Evaluation of the Effectiveness of Xanthine Oxidoreductase Inhibitors on Haemodialysis Patients using a Marginal Structural Model
Source: Sci Rep. 2017 Oct 25;7:14004. doi: 10.1038/s41598-017-13970-4 (PMC5656650; doi:10.1038/s41598-017-13970-4)
Supplement: Supplementary file 1 — Supplementary Information [file 41598_2017_13970_MOESM1_ESM.pdf]

Supplementary Information

Evaluation of the Effectiveness of Xanthine Oxidoreductase Inhibitors on Haemodialysis Patients using a Marginal Structural Model

Takeo Ishii[1,2,*], Masataka Taguri[3], Kouichi Tamura[2,*], and Kunio Oyama[1]

[1] Department of Internal Medicine, Yokohama City, Yokohama-Daiichi Hospital, Yokohama 220-0011, Japan
[2] Department of Medical Science and Cardiorenal Medicine, Yokohama City University Graduate School of Medicine, Yokohama 236-0004, Japan
[3] Department of Biostatistics, Yokohama City University Graduate School of Medicine, Yokohama 236-0004, Japan

***Corresponding authors**
Takeo Ishii
Department of Internal Medicine, Yokohama Daiichi Hospital,
2-5-15 Takashima, Nishi-ku, Yokohama 220-0011, Japan
E-mail: takeo.ishii@grp.zenjinkai.or.jp

Kouichi Tamura
Department of Medical Science and Cardiorenal Medicine, Yokohama City University Graduate School of Medicine,
3-9 Fukuura, Kanazawa-ku, Yokohama 236-0004, Japan
E-mail: tamukou@med.yokohama-cu.ac.jp

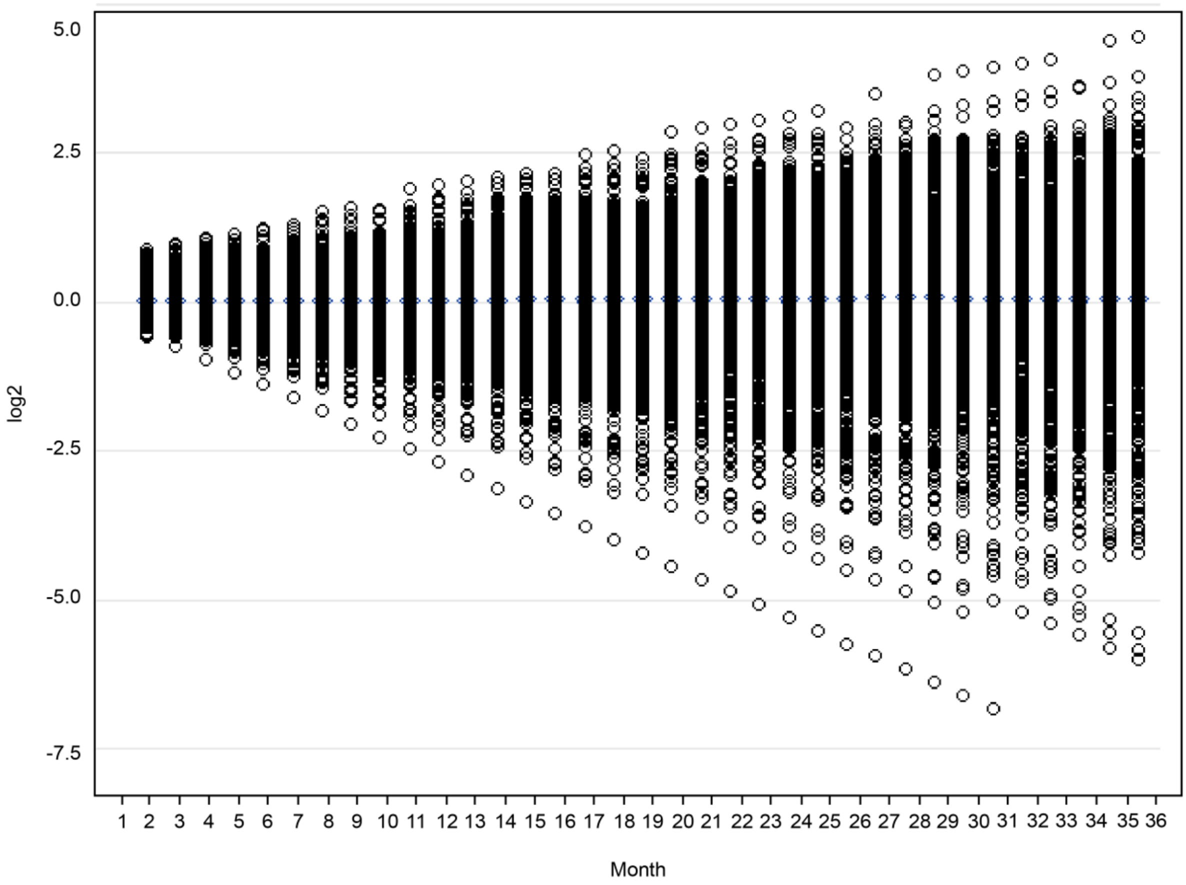

Supplementary Figure S1. Distribution of Stabilized Weights. After the denominator was created (1/ps_pred_n_, 1/(1-ps_pred_n_) and the numerator was created (ps_pred_n_, 1-ps_pred_n_), the distribution of the Stabilized Weight in this figure was created for 36 months of data. The numerator and 1/denominator were multiplied to calculate the stabilized weight (numerator/denominator). Stabilized Weights were calculated into log2 in this figure.

Supplementary Table S1. Cox Hazard Analysis for New-Onset CVD events

Parameter	Hazard Ratio	SE	p	95% CI		*: p<0.05
				Lower Limit	Upper Limit	
**Baseline**						
Age (y)	1.00	0.001	0.41	1.00	1.00	
BMI (kg/m^2)	1.00	0.003	0.47	0.99	1.00	
Sex, male n (%)	0.99	0.028	0.70	0.94	1.05	
DM, yes n (%)	1.07	0.024	0.00	1.02	1.12	*
XORi, yes n (%)	1.02	0.027	0.37	0.97	1.08	
HD vintage (m)	1.00	0.000	0.42	1.00	1.00	
**Baseline Serological Parameters**						
Hb (g/dL)	1.01	0.010	0.49	0.99	1.03	
Fe (μg/dL)	1.00	0.000	0.68	1.00	1.00	
Ferritin (ng/mL)	1.00	0.000	0.29	1.00	1.00	
BUN (mg/dL)	1.00	0.002	0.69	1.00	1.00	
UA (mg/dL)	1.00	0.009	0.98	0.98	1.02	
Cr (g/dL)	0.99	0.011	0.21	0.96	1.01	
Na (mEq/L)	1.00	0.004	0.76	0.99	1.01	
Pi (mg/dL)	1.01	0.010	0.38	0.99	1.03	
Intact PTH (pg/mL)	1.00	0.000	0.50	1.00	1.00	
Alb (g/mL)	1.01	0.034	0.82	0.94	1.08	
B2MG (mg/dL)	1.00	0.002	0.37	1.00	1.01	
TC (mg/dL)	1.00	0.000	0.25	1.00	1.00	
LDL (mg/dL)	1.00	0.001	0.58	1.00	1.00	
HDL(mg/dL)	1.00	0.001	0.41	1.00	1.00	
CGR (%)	1.00	0.001	0.34	1.00	1.00	
**Haemodialysis Related Parameters**						
Delta BW (kg/HD)	1.00	0.012	0.96	0.98	1.02	
SBP (mmHg)	1.00	0.000	0.02	1.00	1.00	*
nPCR (g/kg/day)	1.13	0.159	0.46	0.82	1.54	
KT/V	0.93	0.045	0.10	0.85	1.01	
Heart Rate(bpm)	1.00	0.001	0.38	1.00	1.00	
**Comorbidity at Baseline**						

Infection and parasite	1.03	0.025	0.20	0.98	1.08	
Neoplasms	1.00	0.050	0.97	0.91	1.11	
Endocrine metabolic disorder	1.08	0.116	0.52	0.86	1.35	
Mental disorder	1.01	0.031	0.71	0.95	1.08	
Nervous system disorder	1.02	0.025	0.41	0.97	1.07	
Eye/ear	1.00	0.025	0.95	0.95	1.05	
CVD complications (baseline)	0.90	0.067	0.10	0.79	1.02	
Respiratory system	1.02	0.051	0.66	0.93	1.13	
Digestive system	1.01	0.082	0.90	0.86	1.19	
Skin disorder	1.01	0.033	0.80	0.95	1.08	
Musculoskeletal	1.06	0.027	0.03	1.01	1.12	*
PCKD and congenital disease	1.02	0.034	0.52	0.96	1.09	
Injury	1.05	0.025	0.03	1.00	1.11	*

Legend:
Baseline DM had a risk for new-onset CVD events (HR 1.072), SBP (HR 0.999), musculoskeletal complication (HR 1.060), and injury complications (HR 1.054).
CVD complications (baseline) included heart failure, hypertension, ischaemic heart disease, pulmonary heart diseases, Af and valvular diseases, cerebrovascular diseases, diseases of the arteries, diseases of the veins, hypotension, and others.
Mental disorder and endocrine metabolic disorder were omitted from the analysis.
Endocrine/metabolic disorder and mental disorder were omitted from the analysis.

Supplementary Table S2. In the IPTW analysis, logistic regression for treatment, ps_pred for denominator and numerator.

**Parameter <denominator>**	**Estimate**	**SE**	**p**
Baseline XORi, yes n	19.48	135.8	0.89
Sex, male n	0.01	0.052	0.79
Age (y)	-0.01	0.002	0.00
BMI (kg/m^2)	0.01	0.006	0.01
HD vintage (m)	0.00	0.000	0.40
DM yes n	-0.08	0.045	0.07
Baseline complications			
Infection and parasites	-0.06	0.043	0.15
Neoplasms	-0.15	0.092	0.09
Endocrine metabolic disorder	-0.57	0.163	0.00
Mental disorder	-0.12	0.050	0.02
Nervous system disorder	-0.12	0.044	0.01
Eye/ear	0.10	0.043	0.02
Baseline CVD Complications			
Heart Failure	-0.06	0.051	0.23
Hypertension	0.08	0.060	0.19
Ischaemic Heart Disease	0.06	0.044	0.19
Pulmonary Heart Disasees	-0.13	2425.100	1.00
Af and Vulvular Disases	-0.02	0.070	0.75
Cerebrovascular Diseases	0.06	0.060	0.32
Diseases of the Arteries	-0.02	0.049	0.70
Diseases of the Veins	0.38	0.090	<.0001
Hypotension and Others	-0.19	0.085	0.02
Respiratory system	0.21	0.104	0.04

**Parameter <numerator>**	**Estimate**	**SE**	**p**
Baseline XORi, yes n	19.53	136.7	0.8864
lag_treatments	3.22	0.0367	<.0001
Sex, male n	0.06	0.0419	0.1892
Age (y)	-0.01	0.00172	<.0001
BMI (kg/m^2)	0.01	0.00525	0.0194
HD vintage (m)	0.00	0.000234	0.92
DM yes n	-0.07	0.0418	0.0784
Infection and parasite	-0.05	0.0421	0.2579
Neoplasms	-0.16	0.0895	0.0771
Endocrine metabolic disorder	-0.67	0.1605	<.0001
Mental disorder	-0.13	0.0491	0.0089
Nervous system disorder	-0.12	0.0427	0.0047
Eye/ear	0.13	0.0416	0.0015
Baseline CVD Complications			
Heart Failure	-0.07	0.050	0.15
Hypertension	0.06	0.058	0.32
Ischaemic Heart Disease	0.04	0.043	0.33
Pulmonary Heart Disasees	-0.04	2438.0	1.00
Af and Vulvular Disases	-0.04	0.067	0.51
Cerebrovascular Diseases	0.06	0.059	0.28
Diseases of the Arteries	-0.01	0.048	0.68
Diseases of the Veins	0.40	0.089	<.0001
Hypotension and Others	-0.12	0.082	0.15
Respiratory system	0.26	0.103	0.01

Digestive system	0.24	0.117	0.04	Digestive system	0.29	0.115	0.01
Skin disorder	-0.02	0.064	0.74	Skin disorder	-0.07	0.063	0.25
Musculoskeletal	-0.20	0.069	0.0	Musculoskeletal	-0.11	0.068	0.11
PCKD and congenital disease	0.00	0.058	0.95	PCKD and congenital disease	0.06	0.057	0.31
Injury	0.12	0.042	0.00	Injury	0.10	0.041	0.01
Time-dependent Variables (Repeated Measured)							
lag_treatments	3.03	0.038	<.0001				
lag_ua	-0.24	0.013	<.0001				
lag_alb	0.16	0.061	0.01				
lag_npcr	0.54	0.268	0.04				
lag_bun	-0.01	0.007	0.05				
lag_cr	0.04	0.010	0.00				
lag_cgr	0.93	0.399	0.02				
lga_fe	-0.00	0.001	0.11				
lag_ferr	-0.00	0.000	0.00				
lag_hb	-0.00	0.020	0.91				
lag_na	-0.02	0.007	0.00				
lag_pi	0.02	0.016	0.32				
lag_tc	0.00	0.001	0.21				
lag_hdl	0.00	0.002	0.29				
lag_hr	0.00	0.002	0.72				
lag_b2mg	0.00	0.003	0.88				
lag_ktv	-0.22	0.098	0.02				
lag_ldl	0.00	0.002	0.48				
lag_bp	0.00	0.001	0.59				
lag_pth	0.00	0.000	0.48				
lag_deltabw	0.03	0.019	0.08				

Legend:
All time dependent and time non-dependent variables were added to the denominator covariate.

All time non-dependent variables, except lag_treatment, were added to the numerator covariate.
lag_: previous observation
treatments: XORi use at each observation, and lag_treatments were the XORi used at the previous observation.

Supplementary Table S3. Weight distribution

**Weight**	**Median**	**1 percent point**	**5 percent point**	**Lower Quartiles Point**	**Upper Quartiles Point**	**95 percent point**	**99 percent point**	**Inter quartile range**	**Mean**	**SD**	**Minimum**	**Maximum**
Numerator	1.000000	0.0000000008388526	0.000003880127	0.1237602	1.000000	1.000000	1.000000	0.8762398	0.7087436	0.4348620	0.0000000	1.000000
**Denominator**	1.000000	0.0000000003707435	0.000003469403	0.1130941	1.000000	1.000000	1.000000	0.8869058	0.7089389	0.4351635	0.0000000	1.000000
STABILIZED_WEIGHT	1.000000	0.2630645	0.6955309	1.000000	1.000000	1.945860	3.628771	0.00000001000000	1.114557	0.5831767	0.008891500	21.94657

Legend: Weight (IPTW, ps_weight) was created using the following formula: for patients treated with XORi, ps_weight = 1 / ps_pred, otherwise 1 / (1 – ps_pred)[10,11]. The ps_weight was cumulatively multiplied by 36 visits, defined as the 1/denominator. The numerator was created with the same covariate as the numerator including previous XORi treatment but without the time-dependent variables, which was created using the following formula: for patients treated with XORi, ps_weight = ps_pred, otherwise (1 – ps_pred)[10,11]. (Supplementary Table S2) as those used for the calculation to create the denominator with inversely, but XORi treatment was in the calculation. The numerator was cumulatively multiplied by 36 times as was the denominator. The numerator and 1/denominator were multiplied to calculate the stabilized weight (numerator/denominator). Stabilised weight that was used to fit the outcome model of the MSM.
